# Effect of Amorphous *Halomonas*-PHB on Growth, Body Composition, Immune-Related Gene Expression and *Vibrio anguillarum* Resistance of Hybrid Grouper (*Epinephelus fuscoguttatus* ♀ × *E*. *lanceolatu* ♂) Juveniles

**DOI:** 10.3390/ani14182649

**Published:** 2024-09-12

**Authors:** Wei Xie, Haoran Ma, Meirong Gao, Dongdong Du, Liangsen Liu, Liying Sui

**Affiliations:** 1 Tianjin Key Laboratory of Marine Resources and Chemistry, Tianjin University of Science & Technology, Tianjin 300457, China; 2 Asian Regional Artemia Reference Center, College of Marine and Environmental Sciences, Tianjin University of Science and Technology, Tianjin 300457, China

**Keywords:** *Halomonas*, poly-β-hydroxybutyrate (PHB), grouper, fatty acid, immunity

## Abstract

**Simple Summary:**

Poly-β-hydroxybutyrate (PHB) has proven potential to promote growth and immunity in aquaculture. This study aimed to investigate the effects of adding amorphous *Halomonas*-PHB to the diet for grouper culture. The findings revealed that supplementation with amorphous *Halomonas*-PHB did not significantly improve fish growth performance, but it increased the content of fatty acids, including long-chain unsaturated fatty acids C18:1n9, EPA, and DHA in fish muscle. Additionally, it enhanced the resistance of the fish to *Vibrio anguillarum* possibly by regulating immune-related gene expression in different tissues and organs. These results will contribute valuable insights for the practical application of amorphous *Halomonas*-PHB in aquaculture.

**Abstract:**

Poly-β-hydroxybutyrate (PHB) is a bacterial metabolite produced by bacteria such as *Halomonas* sp. that serves as a carbon and energy storage compound for bacteria under nutrient-limited conditions. Two experiments were conducted to investigate the effects of dietary supplementation with *Halomonas*-PHB on hybrid grouper (*Epinephelus fuscoguttatus* ♀ × *E*. *lanceolatu* ♂). In experiment I, juvenile groupers were fed basal diets supplemented with 3% *Halomonas*-PHB (3% HM-PHB) containing 1.4% PHB and 3% *Halomonas* (3% HM) without PHB, as well as a control diet, for seven weeks. The results showed no significant difference in survival rate, weight gain, and crude fat content between the 3% HM-PHB group and the control group; however, the crude protein of the 3% HM-PHB group was significantly lower than that of the control group. Furthermore, supplementation with 3% HM-PHB increased the fatty acids content in fish muscles, including long-chain unsaturated fatty acids C18:1n9, EPA, and DHA. In experiment II, groupers were fed a basal diet supplemented with 6.5% *Halomonas*-PHB (6.5% HM-PHB) containing 3% PHB and 6.5% *Halomonas* (6.5% HM) containing no PHB, as well as a basal diet (Control). After seven weeks of rearing, the fish were challenged with *Vibrio anguillarum* for 48 h. Although no significant difference in survival rate and growth was observed among different groups, the dietary supplement of 6.5% *Halomonas*-PHB improved the survival rate of *V. anguillarum* challenged grouper and significantly increased the gene expressions of catalase (*CAT*) and superoxide dismutase (*SOD*) in blood, interleukin 1 (*IL1*) and interleukin 10 (*IL10*) in the liver, spleen, head kidney, and blood (*p* < 0.05). In conclusion, dietary supplementation of *Halomonas*-PHB had no significantly positive effect on fish growth performance but increased the content of fatty acids, including long-chain unsaturated fatty acids C18:1n9, EPA, and DHA in fish muscle; it also improved the *V. anguillarum* resistance, possibly through increasing immune-related gene expression in different tissues and organs. Our findings offer compelling evidence that *Halomonas*-PHB can be utilized as a feed additive in intensive grouper farming to enhance the groupers’ resistance to *Vibrio*.

## 1. Introduction

The hybrid grouper (*Epinephelus fuscoguttatus* ♀ × *E*. *lanceolatu* ♂) is a valuable high-grade economic species with significant nutritional value. Farmed groupers production has rapidly increased in China since 2010, reaching an annual output of 205 thousand tons in 2023 [1]. However, disease outbreaks pose a major challenge to the survival and growth of fish. Bacterial, viral, and parasitic infections, including *Vibrio* and *Aeromonas*, iridovirus, and nervous necrosis virus, have frequently occurred in grouper farming [2]. Conventional methods for controlling bacterial diseases in aquaculture include antibiotics, antifungals, and agrochemicals. However, the misuse of these chemicals can lead to bacterial resistance [3,4], environmental pollution, and adverse impacts on human health [5]. Therefore, it is necessary to find alternatives to control the aquatic diseases. Dietary supplement strategies have been extensively studied as effective management measures and have significantly reduced disease risk. The addition of specific nutrients or functional ingredients could strengthen the immune system of fish, thereby increasing its resistance to pathogens and reducing disease-related mortality [6,7].

Poly-β-hydroxybutyrate (PHB) is a carbon and energy storage compound in prokaryotic cells that has shown a potential to enhance the survival, growth, and immunity in fish farming. PHB can undergo partial or complete degradation into β-hydroxybutyric acid monomer in the intestine of aquatic animals. β-hydroxybutyric acid has been observed to increase bacterial richness and change bacterial community in the intestine [8,9]. Moreover, elevated levels of short-chain fatty acids (SCFAs) resulting from PHB degradation supported a lower pH environment while binding calcium cations within the intestine and improving intestinal health to promote mineral availability [10]. Conversely, studies have indicated that PHB can boost the immune power and enhance resistance against pathogens [11,12]. SCFAs derived from PHB degradation can traverse the bacterial cell wall and reduce cytoplasmic pH in pathogenic cells, thereby diminishing their infectivity [13]. Additionally, SCFAs in the intestine provide an additional energy substrate for leukocytes and other immune cells to boost immunity [14]. 

Bacteria of the *Halomonas* genus generally exhibit a wide tolerance to salinity variations. Several *Halomonas* species have demonstrated the ability to accumulate PHB in their cells, reaching up to 80% cell dry weight (cdw), when provided with additional carbon sources, such as glucose, xylose, sucrose, maltose, sodium acetate, and butyric acid [15,16,17]. Two forms of PHB are amorphous and crystalline, and amorphous PHB has more pores and lower crystallinity, contributing to its better absorption characteristics [18,19]. Compared to the crystalline-form PHB that is solvent-extracted from bacteria, the amorphous bacterial PHB is easier to degrade in the gut and is utilized by aquatic animals [20,21,22]. In this study, PHB-accumulating *Halomonas* was fermented, and the effect of *Halomonas*-PHB on the growth, body composition, gut tissue structure, and resistance against *Vibrio* were investigated in juvenile groupers. This study aims to evaluate the potential application of *Halomonas*-PHB in intensive grouper farming.

## 2. Material and methods

### 2.1. Halomonas Fermentation 

The *Halomonas* strain (CGMCC No.13730) was subjected to fermentation in the culture medium containing 10 g/L yeast extract, 7.5 g/L acid hydrolyzed casein, and 30 g/L glucose to promote the accumulation of PHB in the cells. In contrast, *Halomonas* without a PHB accumulation was cultured in a CM medium without glucose supplementation. The fermentation process was conducted using 5-L fermentation equipment (Biotech, Shanghai, China) at 37 °C, pH 7.5, and with agitation speed set between 200–400 rpm. The PHB content in *Halomonas* cells was determined by HPLC [21]. Specifically, the PHB content in PHB-accumulating *Halomonas* was 46.5% on cell dry weight, while no PHB was detected in *Halomonas* cultured without glucose supplementation.

### 2.2. Feed Preparation and Experimental Design

*Halomonas* pellet was sprayed on the surface of the commercial pelleted feed (Ezukeeru, Three Links Central Feed (Shandong) Co., Ltd., Weifang, China) to supplement *Halomonas* and *Halomonas*-PHB in the feeds. The control feed was sprayed with equivalent sterile water. The diets were dried under cold air and stored at −20 °C. The crude protein and crude lipid content in the diets were determined using the Kjeldahl method (2300-Kjeldahl apparatus, FOSS, Copenhagen, Denmark) and Soxhlet extraction, respectively [23]. The fatty acid methyl esters (FAME) profile was analyzed by GC-MS [24] (Table 1). The hybrid groupers, *Epinephelus fuscoguttatus* ♀ × *E. lanceolatu* ♂, were obtained from Hebei Xinhai Aquatic Biotechnology Co. Ltd., Huanghua, China, and were raised in the lab to acclimate for one week. 

#### 2.2.1. Experiment I 

In experiment I, groupers (2.92 ± 0.08 g) were divided into 12 tanks (four replicates per diet, with a total of 30 fish in each tank) containing 70-L of diluted brine (salinity of 25 g/L) equipped with a recirculation system. The fish were fed three diets: commercial feed (Control), feed with 3% *Halomonas* (3% HM, 0% PHB), and feed with 3% *Halomonas*-PHB (3% HM-PHB, 1.4% PHB). During the 7-week feeding trial, the diets were given to fish *ad libitum* three times a day to apparent satiety. The fish were reared in the recirculating aquaculture system in which the water parameters were maintained at 28 ± 1 °C, dissolved oxygen 7.0 ± 0.5 mg/L, and pH 7.5–7.8. 

#### 2.2.2. Experiment II

Based on the results of experiment I, in experiment II, the dietary supplementation of *Halomonas* and *Halomonas*-PHB was increased to 6.5% *Halomonas* (6.5% HM, 0% PHB) and 6.5% *Halomonas*-PHB (6.5% HM-PHB, 3% PHB) to explore the effect on immune function. The initial fish body weight was 1.31 ± 0.08 g. The rearing conditions were the same as described in experiment I.

### 2.3. Sample Collection and Parameter Determination

#### 2.3.1. Growth Performance 

At the end of each experiment, the growth performance was assessed as follows:

Survival rate (SR, %) = (final fish number/initial fish number) × 100

Weight gain rate (WGR, %) = (final body weight − initial body weight)/initial body weight × 100

Specific growth rate (SGR %/d) = [ln (final body weight) − ln (initial body weight)]/days × 100

Feed conversation ratio (FCR) = Feed intake/(final body weight − initial body weight)

Hepatosomatic index (HSI, %) = (individual liver weight/final body weight) × 100

#### 2.3.2. Fish Muscle Composition and Histology Analysis of Intestine in Experiment I

At the end of the 7-week rearing, three groupers from each tank were randomly sampled and dissected after anesthesia with 0.01% MS-222. The crude protein, crude lipid, and fatty acid content in muscle were obtained and detected by the methods given in Section 2.2. The midguts of three groupers were immersed and fixed overnight in 4% paraformaldehyde at 4 °C, were subsequently dehydrated by a 70%, 80%, 95%, and 100% ethanol solution, respectively, and were then transparent with xylene and embedded in paraffin. The paraffin sections of the intestine were cut in a microtome at 3 μm thickness and stained with hematoxylin-eosin (HE). The stained slides were examined and imaged under an inverted microscope (Nikon Eclipse Ti, Tokyo, Japan). The organizational structure of each slide in the intestine was analyzed by Image-J V1.53t. 

#### 2.3.3. Vibrio Culture and Challenge

*Vibrio anguillarum* strain (MCCC 1A07299) was obtained from the Third Institute of Oceanography of State Oceanic Administration, Qingdao, China. The bacteria were cultured at 28 °C for 24 h in a 216 L medium containing 1.0 g/L sodium acetate, 10.0 g/L peptone, 2.0 g/L yeast, 0.5 g/L trisodium citrate, and 30 g/L NaCl. *V. anguillarum* cells were collected at 3000 rpm for 10 min. The pellet was rinsed and re-suspended with sterilized 0.9% physiological saline water. 

In experiment II, after a 7-week culture, 36 groupers were randomly selected from each treatment and challenged with *V. anguillarum*. According to the preliminary test results and previous research [25], each fish was intraperitoneally injected with 0.1 mL *V. anguillarum* suspension (1.0 × 10^8^ cfu/mL), and 0.1 mL physiological saline water was injected in the control group. The mortality of the groupers was determined within 48 h. 

#### 2.3.4. Immune-Related Gene Expression

After 48 h challenge with *V. anguillarum*, three groupers were randomly collected from each replicate tank and anesthetized with 0.01% MS-222. The liver, spleen, head kidney, and blood were dissected after anesthesia, frozen immediately with liquid nitrogen, and stored at −80 °C. Total RNA was extracted using Trizol Reagent (Tiangen Biotech, Beijing, China) following the manufacturer’s protocol. The quantity and quality of the extracted RNA was determined using a bio-photometer (Eppendorf, Hamburg, Germany) and on 1.2% agarose gel electrophoresis. RNA was reverse transcribed using Prime Script™ RT-PCR Kit (TaKaRa, Shiga, Japan). The specific primer pairs were chosen based on the references (Table 2). The β-actin gene was used as a housekeeping gene. The qPCR was amplified (Bio-Rad, Heracles, CA, USA) using SYBR Premix Ex Taq (TaKaRa, Shiga, Japan). Before the qPCR, the specificity and amplification efficiency of the primers were detected. A total volume of 20 μL reaction mixtures contained 2.4 μL cDNA sample, 10 μL 2 × SYBR Premix Ex Taq, 0.8 μL each of the 10 μM forward and reverse primers, and 6 μL ddH_2_O. The PCR conditions were as follows: 95 °C for 30 s, then 40 cycles at 95 °C for 10 s, followed by different annealing temperatures for 30 s. All samples were run in three triplicates. The threshold cycle (Ct) values were obtained from each sample. Relative gene expression levels were evaluated using the 2^−ΔΔCT^ method [26].

### 2.4. Statistical Analysis

All data was analyzed using SPSS (version 20.0) software and expressed as mean ± SD when there were replicates. All the data were tested for normality, homogeneity, and independence before ANOVA. Significant differences among the treatments were determined using one-way ANOVA followed by Duncan’s test at *p* < 0.05. 

## 3. Results

### 3.1. Growth Performance

In both experiments, the survival rates of the groupers were above 93%, there was no negative effect on growth, and no disease symptoms were observed during 7-week rearing. The final fish body weight in the 3% HM group was significantly higher than the control and 3% HM-PHB group (*p* < 0.05) (Table 3). No significant difference was observed in the WG, SGR, and FCR of the three groups (*p* > 0.05). 

### 3.2. Muscle Nutritional Composition

In experiment I, the crude fat content in fish muscles ranged from 6.92% dw to 8.59% dw, and there was no significant difference among the groups (*p* > 0.05) (Table 4). The crude protein content in fish muscles ranged from 77.87% dw to 84.22% dw, significantly lower in 3% HM and 3% HM-PHB compared to the control group. 

The content of C14:0, C16:0, C16:1n7, C17:0, C18:0, C18:1n9, C18:1n7, 18:2n6, and 20:1n9 in fish muscles of 3% HM-PHB group were significantly higher than the control group, while 3% HM group was in between (*p* < 0.05) (Table 4). Although there was no significant difference (*p* > 0.05), the content of EPA and DHA in the 3% HM-PHB group was higher than in the control and 3% HM groups. 

### 3.3. Histological Structure of Intestine

**Figure 1:**
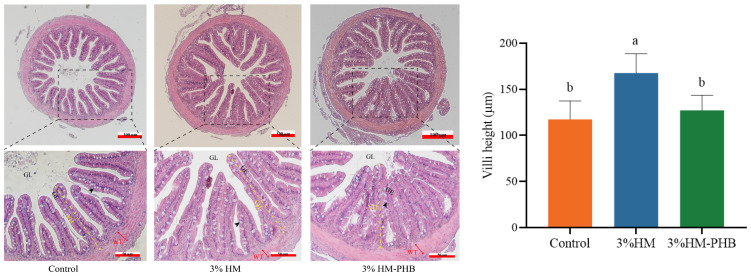
HE-stained midgut tissue of grouper fed the experimental feeds for seven weeks. (A) Control group; (B) 3% HM group; (C) 3% HM-PHB. VH, villi height; GE, gut epithelium; GL, gut lumen; WT, wall thickness; black arrow, goblet cells. Different superscript letters represent significant differences (*p* < 0.05).

The intestinal tissue structure of the three groups was normal and undamaged, indicating that PHB has no negative impact on the intestine (Figure 1). A significant histological change was observed in the villi height in the 3% HM group, which was significantly higher than in other groups. There was no significant difference between the PHB group and the control group.

### 3.4. Resistance to Vibrio Challenge

**Figure 2:**
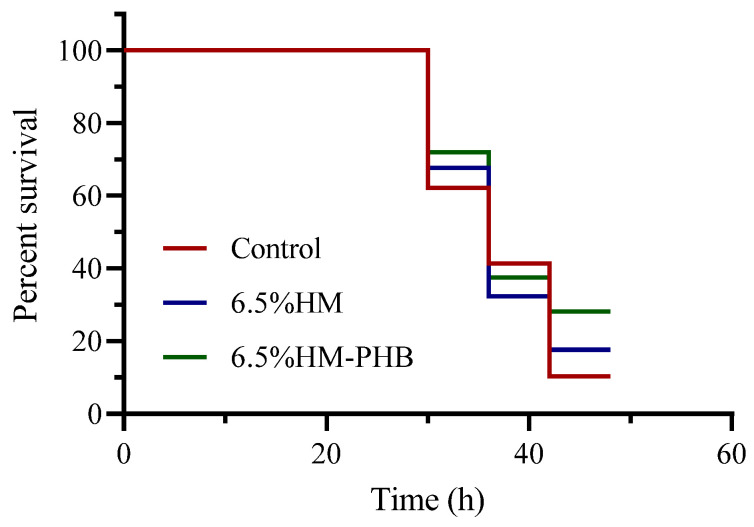
The survival rate of the groupers challenged by *V. anguillarum* for 48 h.

In experiment II, groupers were challenged with *V. anguillarum* after 7-week rearing. The fish exhibited slower swimming, and the survival rate of groupers declined gradually after 24 h of exposure to *V. anguillarum*. The yellow-green liquid in the intestine and bleeding symptoms were observed in dead fish. At 48 h, the survival rate of the control, 6.5% HM, and 6.5% HM-PHB groups were 9.1%, 16.7%, and 25.7%, respectively (Figure 2). 

### 3.5. Antioxidative Enzyme and Immune-Related Gene Expression

**Figure 3:**
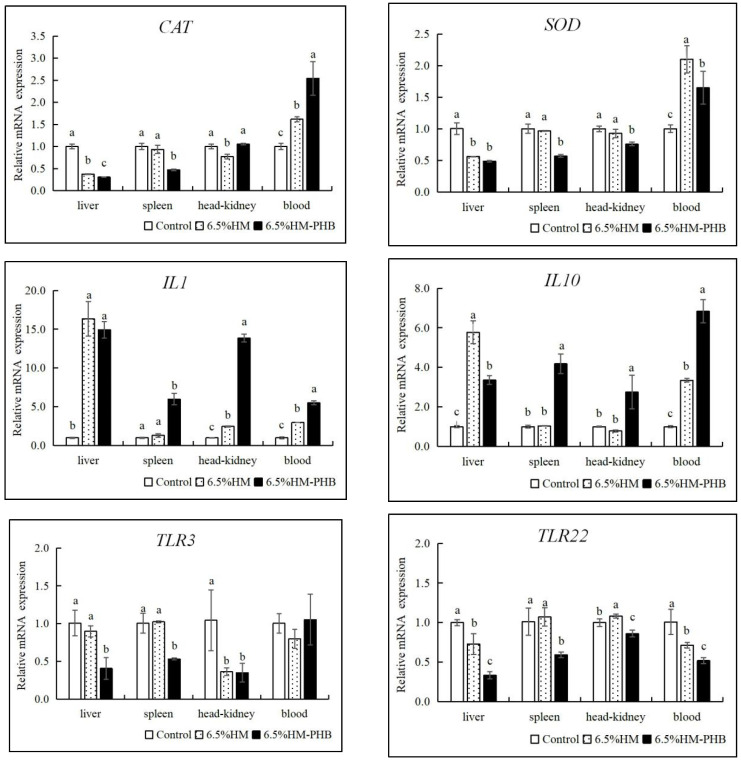
Relative expression levels of antioxidation and immune-related genes in different tissues of groupers fed different diets for seven weeks and challenged by *V. anguillarum* for 48 h. Different superscript letters represent significant differences (*p* < 0.05).

In experiment II, after exposure to *V. anguillarum* for 48 h, the antioxidative enzyme gene *CAT* and *SOD* expression in fish blood were significantly upregulated in both the 6.5% HM-PHB and 6.5% HM group compared to the control group (*p* < 0.05) (Figure 3). Moreover, there was a significant improvement in the gene expression of inflammatory factor *IL1* and anti-inflammatory *IL10* in the fish spleen, head kidney, and blood in the 6.5% HM-PHB group compared to both the 6.5% HM group and the control (*p* < 0.05), except for *IL1* expression in the liver which showed similar levels between the 6.5% HM-PHB and 6.5% HM group (*p* > 0.05). Additionally, *IL10* exhibited significantly higher expression in the 6.5% HM group than in the 6.5% HM-PHB group (*p* < 0.05). The *TLR3* and *TLR22* gene expression were significantly lower in fish liver, spleen, head kidney, and blood samples from the 6.5% HM-PHB group than the 6.5% HM group and the control (*p* < 0.05). 

## 4. Discussion 

PHB, a bacterial compound serving as an energy and carbon storage source, has been considered a potential biocontrol agent in aquaculture due to its ability to enhance the growth, immune, and antioxidant ability of aquatic animals [30,31]. As a lipid-soluble short-chain fatty acid polymer, PHB can be partially degraded into β-hydroxybutyrate monomer in the intestine of aquatic animals and is utilized by intestinal microorganisms and epithelial cells [11]. β-hydroxybutyrate acid exhibits diverse biological functions, including anti-inflammatory and antioxidant properties [32,33]. Previous studies have shown the positive effect of PHB on aquaculture species. The dietary supplementation of PHB particles significantly improved crustaceans’ growth performance by improving feed utilization, intestinal digestive function, and gut microbiota status [32,34]. However, the effects of PHB varied among different fish species. For example, while PHB exhibited beneficial effects on European sea bass (*Dicentrarchus labrax*) growth, it did not show similar results for Siberian sturgeon (*Acipenser baerii*) [8,35]. In our study, 3% HM-PHB (containing 1.4% PHB) increased the growth performance in hybrid grouper, but the effect was not significant (*p* > 0.05). This discrepancy may be attributed to physiological differences between species, variations in experimental conditions, and/or the specific form of PHB. Unlike commercial products of purified PHB polymers, the whole cell *Halomonas*, including 46.5% PHB of cell dry weight, was used in our study. It has been claimed that bacterial-storage amorphous PHB granules are more effective than solvent-extracted crystalline PHB, as the former is easier to degrade in the gut of aquatic animals [20,21,22]. In this study, no significant differences in FCR values were observed among the groups, consistent with the findings of WGR and SGR, indicating that PHB had no discernible impact on feed utilization under these experimental conditions. However, it is noteworthy that all groups exhibited FCR values below 1, suggesting efficient feed conversion. This could be attributed to several factors within the overall experimental setup, including optimized feeding strategies, low stocking density, and a recirculating aquaculture system (RAS) environment. Previous research has demonstrated that increasing feeding frequency can improve FCR, particularly for small fish [36]. Additionally, maintaining an appropriate stocking density helps prevent stress caused by overcrowding or high density and contributes to lower FCR values [37]. Furthermore, combining a lower stocking density with an RAS system facilitates achieving low FCR values as water reuse reduces consumption and enhances nutrient recycling [38].

In the HM-PHB group, crude protein levels significantly decreased, indicating that *Halomonas*-PHB affects protein metabolism and utilization. Considering that intestinal digestion and absorption are crucial initial steps in nutrient utilization, it is plausible that PHB’s impact on intestinal digestive enzymes could potentially alter protein absorption. Studies have shown that PHB affects intestinal digestive enzymes in fish, which vary according to species [39,40]. Therefore, it is necessary to investigate further the effects of *Halomonas*-PHB on intestinal digestive enzyme activity and amino acid absorption in grouper to clarify the mechanism by which PHB influences protein deposition efficiency. On the other hand, the muscle protein content of the 3% HM group was significantly higher than that of the HM-PHB group. This observation may be attributed to enhanced nutrient absorption in the fish intestine, as evidenced by the increased villi height observed specifically in the 3% HM group. In addition, *Halomonas* was a dominant protease-producing bacterium in carnivorous fish, which might be involved in protein digestion [41].

The presence of higher levels of long-chain unsaturated fatty acids in muscles is associated with enhanced nutritional value of the fish. In our study, PHB supplementation in diet increased the PUFA content in fish muscle. Dietary PHB supplements could increase the total fat content by 10% in tilapia *Oreochromis niloticus* [42]. These results are possibly due to the metabolism and transformation of PHB derived from feed, which likely contributes to the synthesis of unsaturated fatty acids in muscles. Moreover, PHB polymers can undergo degradation into oligomers and hydroxybutyric acid in the intestine [43,44]. As a SCFA, β-hydroxybutyric acid serves as an energy source for organs and tissues during metabolic processes. Research has indicated that feed supplemented with PHB improves weight gain and SCFA production in the gut of aquatic animals, thereby meeting energy demands and promoting intestinal absorption [8,35]. Taken together, these pieces of evidence provide a plausible explanation for the observed increase in PUFA content within fish muscles.

Vibriosis is a prevalent disease in grouper farming [2]. Pathogen infection can induce oxidative stress and activate the antioxidative response in aquatic animals. It has been reported that the pathogenic infection increased in leucocytes in the blood, resulting in inflammatory reactions that generated excessive reactive oxygen species (ROS) during phagocytosis or respiratory oxidative burst [45,46]. Maintaining an appropriate level of ROS is crucial for effectively eliminating harmful pathogens; however, an imbalance with excessive ROS can lead to oxidative stress [47]. Therefore, the antioxidant enzymes, such as CAT and SOD, could be used to maintain a balance of oxidative and antioxidant homeostasis during infection [48]. In this study, the supplement of PHB enhanced the expression of *SOD* and *CAT* genes in fish blood upon the *Vibrio* challenge, indicating that *Halomonas*-PHB can improve the antioxidative capacity of groupers to restore the redox imbalance caused by infection. Similar results have been reported that PHB stimulates the soiny mullet *Liza. haematocheila* oxidation system and that a 2% dietary PHB can increase the total antioxidant capacity, as well as the CAT and SOD activity in serum [49]. 

Aquatic animals mainly depend on the innate immunity modulated by pattern recognition receptors (PRR). Toll-like receptors (TLRs) play a crucial role as pivotal PRRs in initiating cellular signaling cascades, thereby triggering the production of diverse cytokines. Studies have found that *TLR3* and *TLR22* are also involved in defending pathogenic microorganisms, with their expression levels being up-regulated after bacterial infection [50,51]. In this study, the *V. anguillarum* challenge significantly decreased the *TLR3* and *TLR22* gene expression level in the 6.5% *Halomonas*-PHB group, indicating that the pathogen was not virulent enough to activate *TLR3* and *TLR22*. This downregulation of gene expression might be due to PHB’s ability to aid in pathogen clearance, as previous studies have reported that 3-HB improved hemocyte phagocytosis and suppressed pathogen growth in shrimp [52]. The anti-inflammatory activity of PHB has been demonstrated, indicating its critical role in modulating immune responses by upregulating the mRNA expression of anti-inflammatory factors [53]. IL1 acts as a pro-inflammatory cytokine that is important for fish disease resistance [54], while IL10 acts as a crucial anti-inflammatory cytokine that can counteract the production of pro-inflammatory cytokines, thereby restraining inflammatory responses [29]. In our study, *Halomonas*-PHB was found to significantly upregulate the gene expression of *IL1* and *IL10* in various tissues following challenge, indicating its ability to modulate the inflammatory response in grouper and enhance the host resistance. The positive evidence regarding the effects of *Halomonas*-PHB on improving muscle quality and boosting immune capacity supports its practical application as an immune enhancer rather than a growth enhancer. Further research is needed to investigate the optimal supplementary level of PHB in combination with feed nutrition for enhancing fish growth and immunity.

## 5. Conclusions

Dietary *Halomonas* and *Halomonas*-PHB had no significant effect on the survival and growth of grouper; however, they led to an increase in the content of long-chain unsaturated fatty acids and improved muscle quality. Moreover, *Halomonas*-PHB exhibited immunostimulatory effects on grouper by upregulating antioxidant genes CAT and SOD, as well as immune-related genes *IL1* and *IL10*, thereby enhancing resistance against *Vibrio* challenge. Consequently, *Halomonas*-PHB holds promise as a potential biological control agent for application in grouper farming. Future research and practical applications should focus on exploring the utilization of *Halomonas*-PHB in high-incidence disease or immunosuppressive scenarios to maximize its efficacy as biological control while optimizing health benefits.

## Figures and Tables

**Table 1:** Crude protein, crude fat content, and fatty acid profile of the experimental feeds.

		Control	3% HM	3% HM-PHB
Crude fat (% dw)		10.11 ± 0.09	9.93 ± 0.03	10.07 ± 0.10
Crude protein (% dw)		55.73 ± 0.15	55. 40 ± 0.16	55.31 ± 0.22
Fatty acid (mg/g dw)	C14:0	6.35	6.24	5.79
	C15:0	0.31	0.32	0.30
	C16:0	28.97	29.04	27.51
	C16:1n7	3.87	3.95	3.71
	C17:0	0.18	0.18	0.19
	C18:0	20.78	20.30	19.78
	C18:1n9	10.54	10.48	10.12
	C18:1n7	5.18	5.43	5.17
	C18:2n6	6.49	6.36	6.30
	C18:3n3	3.06	3.05	3.08
	C20:1n9	2.28	2.24	2.43
	C20:5n3 (EPA)	14.72	14.40	14.32
	C22:6n3 (DHA)	12.12	11.76	11.50

Note: dw, dry weight; EPA, eicosapentaenoic acid; DHA, docosahexaenoic acid.

**Table 2:** The primer sequences of the immune-related gene for qPCR analysis.

Primers	Primers Forward/Reverse (5′ to 3′)	Amplification Efficiency	Annealing Temperature	References
Catalase (*CAT)*	F: GCGTTTGGTTACTTTGAGGTGA R: GAGAAGCGGACAGCAATAGGT	97.9%	60	[27]
Superoxide dismutase (*SOD*)	F: TACGAGAAGGAGAGCGGAAGA R: ATACCGAGGAGGGGGATGA	90.7%	60	[27]
Interleukin 1 (*IL1*)	F: CGACATGGTGCGGTTTC R: TCTGTAGCGGCTGGTGG	109.2%	60	[28]
Interleukin 10 (*IL10*)	F: TTCGACGAGCTCAAGAGTGAG R: TGCCGTTTAGAAGCCAGATACA	109.6%	64	[29]
Toll-like receptor 3 (*TLR3*)	F: TCTCCATTCCGTCACCTTCC R: TCATCCAGCCCGTTACTATCC	103.3%	64	[29]
Toll-like receptor 22 (*TLR22*)	F: CGAGCCAGGTAAACCCATCA R: CTCATCAAACAGGCGGAAGC	92.1%	60	[29]

**Table 3:** Growth performance of groupers fed different diets for seven weeks.

Treatment	Control	3% HM	3% HM-PHB
SR (%)	97.5 ± 3.19	100	100
IBW (g)	2.93 ± 0.03	2.97 ± 0.04	2.85 ± 0.09
FBW (g)	32.71 ± 0.86 ^b^	37.8 ± 2.40 ^a^	35.23 ± 1.96 ^ab^
WG (%)	1015.41 ± 83.16	1172.28 ± 84.91	1140.85 ± 116.50
SGR (%/day)	4.93 ± 0.13	5.22 ± 0.15	5.08 ± 0.13
FCR	0.76 ± 0.02	0.78 ± 0.01	0.80 ± 0.02
HSI (%)	3.31 ± 0.36	3.17 ± 0.34	3.02 ± 0.22

Different superscript letters represent significant differences (*p* < 0.05). SR, survival rate; IBW, initial body weight; FBW, final body weight; WG, weight gain; SGR, specific growth rate; FCR, feed conversation ratio; HSI, hepatosomatic index.

**Table 4:** Crude protein, crude fat content, and fatty acid profile in the muscle of groupers fed different diets for seven weeks.

		Control	3% HM	3% HM-PHB
Moisture (%)		73.50 ± 0.46 ^a^	74.41 ± 0.72 ^ab^	72.81 ± 1.08 ^b^
Crude fat (% dw)		6.92 ± 1.03	8.59 ± 1.58	7.62 ± 1.32
Crude protein (% dw)		84.22 ± 2.00 ^a^	82.77 ± 2.89 ^a^	77.87 ± 3.21 ^b^
Fatty acid (mg/g dw)	C14:0	1.96 ± 0.98 ^b^	3.05 ± 0.32 ^ab^	3.46 ± 0.93 ^a^
	C15:0	0.21 ± 0.08	0.28 ± 0.03	0.31 ± 0.05
	C16:0	10.75 ± 4.41 ^b^	16.63 ± 2.10 ^ab^	19.06 ± 4.53 ^a^
	C16:1n7	2.42 ± 1.08 ^b^	3.82 ± 0.39 ^ab^	4.45 ± 1.14 ^a^
	C17:0	0.16 ± 0.06 ^b^	0.22 ± 0.02 ^ab^	0.24 ± 0.04 ^a^
	C18:0	3.46 ± 1.14	4.40 ± 0.53	4.93 ± 0.92
	C18:1n9	6.65 ± 2.71 ^b^	10.18 ± 1.44 ^ab^	11.84 ± 2.84 ^a^
	C18:1n7	1.58 ± 0.59 ^b^	2.17 ± 0.09 ^ab^	2.47 ± 0.56 ^a^
	C18:2n6	2.85 ± 1.10 ^b^	3.96 ± 0.33 ^ab^	4.39 ± 0.86 ^a^
	C18:3n3	0.88 ± 0.34	1.13 ± 0.15	1.14 ± 0.24
	C20:1n9	0.81 ± 0.27 ^b^	1.19 ± 0.13 ^ab^	1.33 ± 0.31 ^a^
	C20:5n3 (EPA)	4.28 ± 1.85	5.76 ± 0.56	6.38 ± 1.13
	C22:6n3 (DHA)	8.73 ± 3.33	10.82 ± 0.94	11.78 ± 1.90

Different superscript letters represent significant differences (*p* < 0.05). dw, dry weight; EPA, eicosapentaenoic acid; DHA, docosahexaenoic acid.

## Data Availability

The raw data supporting the conclusions of this article will be made available by the authors on request.
